# A database of geomagnetic observatory monthly means: from historic to the satellite era

**DOI:** 10.1186/s40623-025-02316-4

**Published:** 2025-12-06

**Authors:** William Brown, Susan Macmillan, Eleanor Maume, Eliot Eaton

**Affiliations:** British Geological Survey, The Lyell Centre, Edinburgh, EH14 4AP UK

**Keywords:** Geomagnetism, Geomagnetic observatory, Secular variation, Historic data, Digitisation

## Abstract

This work brings together the contents of previously disparate databases of absolute ground geomagnetic observations, both historic and ongoing, to process the data consistently into a coherent and accessible format for researchers. This includes converting reported time resolutions and coordinate systems into a uniform format, and applying all documented observatory baseline changes to the data records, to create a single unified vector time series of monthly mean values for each geomagnetic observatory. The data are made available freely and anonymously to researchers via a web service, updated on a monthly basis with the latest definitive and non-definitive observations reported to the World Data Centre for Geomagnetism (Edinburgh) and INTERMAGNET or Tromsø Geophysical Observatory, respectively. The current database covers 327 observatories worldwide, with observations from 1883 up until present. A separate effort involved digitising tables of monthly means in the 1841–1925 yearbooks of the Royal Observatory, Greenwich, United Kingdom and converting to homogeneous units. The resulting data represent some of the earliest magnetic data at monthly time resolution and may be of use to future studies. However, they cannot be incorporated into the monthly means database as the field vector information is incomplete; we publicise them here instead.

**Graphical Abstract:**

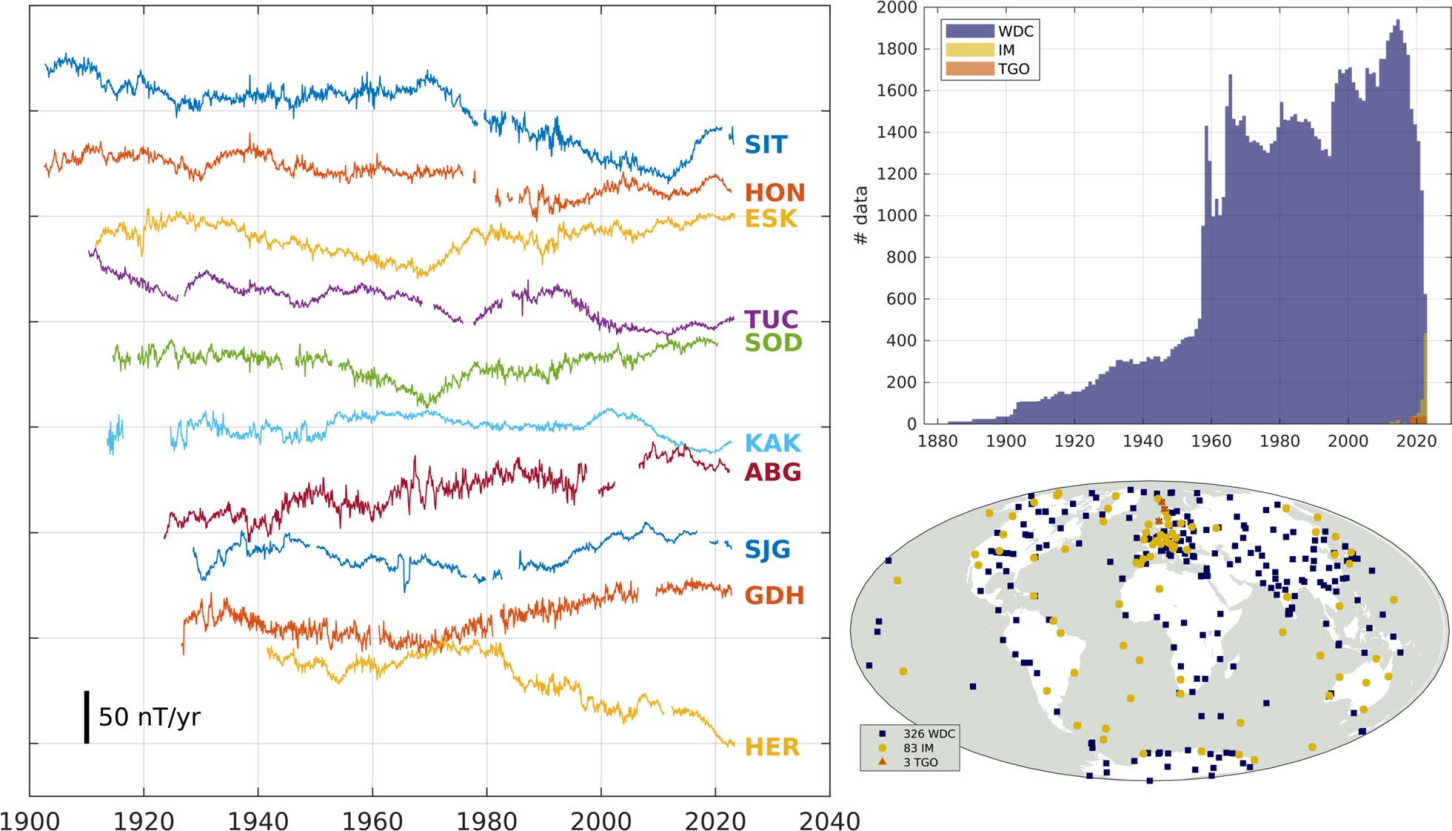

## Introduction

Observatory data have historically been the primary source of direct measurement of the geomagnetic field. The long-term time variations of the geomagnetic field—the secular variation (SV)—can be directly observed at such fixed locations, and continuous series are available over long periods, in some cases over a century. These data are a vital resource for historical information about the geomagnetic field, and continue to provide essential information even in the age of dedicated satellite-based measurements, as the only direct observations of SV. As recently as the period between September 2010 and November 2013, when no dedicated geomagnetic satellite was in orbit, the ground observatory network was relied upon for global magnetic observations; such an occurrence is possible again if there is no long-term successor to the European Space Agency’s Swarm mission.

The World Data Centre for Geomagnetism, Edinburgh (hereafter the WDC, British Geological Survey 2022c), has hosted the master catalogue since 2007, and holds minute, hour and annual mean data. Data are submitted to the WDC by observatory operators once definitive values have been obtained. Observatories meeting prescribed standards may also report data at various intermediate (non-definitive) stages of processing to INTERMAGNET (INTERMAGNET 2022), at second and minute sampling rates, with the aim of achieving real-time delivery. There are also observatories not affiliated with INTERMAGNET which report non-definitive observatory data in real time, such as those reporting to Tromsø Geophysical Observatory (hereafter TGO, Tromsø Geophysical Observatory 2022). Observatory data are generally reported as mean values at sampling times of a year, an hour, a minute, or a second. Parc St Maur (PSM), France is the site providing the earliest hourly data in the WDC, for 1883 (see Fouassier and Chulliat 2009), though the WDC contains annual means from 70 other sites between 1813 and 1883, inclusive. Dumont d’Urville (DRV), Antarctica and Port-aux-Francais (PAF), French Southern Territories are the sites providing the earliest minute mean data in the WDC, starting in 1969.

Monthly data are often used in studies of SV, particularly the rapid signals of geomagnetic jerks (see, e.g., Mandea et al. 2010; Brown et al. 2013), and field modelling (e.g., Finlay et al. 2020). Monthly means provide a convenient resolution for many studies; long enough to reduce the effects of the more rapidly varying external field sources, such as the magnetosphere and ionosphere, but short enough to reveal the relatively slower changes of the core field in detail. Often the values are computed by the authors of a given study from various collections of data at 1 min or hourly sampling rates, and using various approaches. Previous public databases of monthly means have existed, most recently that run by the Bureau Central de Magnétisme Terrestre (BCMT, Chulliat and Telali 2007). Support for this service ceased in 2017 and recently the British Geological Survey (BGS) agreed to take on responsibility for the endeavour.

In the following sections, we describe the process of producing a database of monthly mean values for the contents of the WDC, supplemented with more recent non-definitive data from INTERMAGNET and TGO where available. First, we describe our data sources, before detailing how we process the data. We then describe the data file format and how to access the database. We discuss our efforts and their results, as well as an effort to digitise historical monthly data from the Royal Observatory, Greenwich, before summarising the main aspects of this work.

### Data sources

We primarily use two cadences of data, from three sources: definitive hourly mean data from the World Data Centre for Geomagnetism, Edinburgh (British Geological Survey 2022c), and quasi-definitive (QD) minute mean data from INTERMAGNET (INTERMAGNET 2022) and Tromsø Geophysical Observatory (Tromsø Geophysical Observatory 2022). The former provides annual data files for each observatory in WDC format (British Geological Survey 2025a) which report data to 1 nT (nanoTesla), or 1’ (minute of angular arc) precision only. The latter provide daily data files for each observatory in IAGA2002 format (IAGA Div. V DAT 2016), which can accommodate data reported to instrument precision in fractions of nT or arc-minutes. To be of use for many types of geomagnetic analyses, for example, global modelling, continuous time-series are desirable. As data reported in short time span files, the contents of many files must be stitched together.

We note that geomagnetic observatory data are also available from other World Data Centres (e.g., Graduate School of Science 2022; Geophysical Center of the Russian Academy of Sciences 2021). We chose to use data from the WDC, Edinburgh as the primary catalogue of definitive hourly data due to it being the database with widest geographic and historic extent. The WDC also permits programmatic access to both data and metadata, allowing the practicality of automation of the database collation on a regular basis. We supplement definitive hourly data with minute data from INTERMAGNET and TGO specifically for the case of quasi-definitive data with the aim of creating the most historically complete and ongoing up-to-date database possible. In this context, definitive data are the final adopted values and quasi-definitive data are those reported within 3 months of measurement using provisional baselines, with the aim of 98% of the data being within 5 nT of the final definitive values (Peltier and Chulliat 2010; Clarke et al. 2013). Definitive data are typically reported after the end of the calendar year when a final annual baseline can be established for an observatory, but completion of this process, submission to and entry into the WDC catalogue can take much longer in many cases. Quasi-definitive data are, in some cases, submitted to INTERMAGNET as quickly as the day following recording.

For speed, we access internally held files at BGS which support the publicly viewable WDC catalogue, as well as the WDC metadata web service (British Geological Survey 2025b). We use the INTERMAGNET web service also hosted by BGS (British Geological Survey 2022b) to access the QD data files. TGO data are accessed via HTTP requests to the TGO RESTful service (Tromsø Geophysical Observatory 2022).

The compilation of definitive and quasi-definitive data to give an up-to-date series for each observatory is not a new concept. Macmillan and Olsen (2013) presented the compilation of hourly mean observatory data for a Level 2 product supporting the European Space Agency (ESA) Swarm mission (AUX_OBS_2), which is also produced by BGS on a quarterly basis. Where AUX_OBS_2 focuses on providing data that are manually quality-controlled and formatted to be used directly as input to or as ground-truth for field model products from the Swarm mission, the database described here aims to provide the full contents of the WDC, in as complete and up-to-date a form as possible, in a consistent and usable form for geomagnetic researchers. It also removes some of the burden of knowledge required to process observatory data from researchers, allowing them to focus on performing their research and not learning how to convert raw observatory files into the required time-series format. We also aim to use a process which is as objective and automated as possible and can be run frequently to keep the database contents updated with minimal effort.

Once the magnetic data values have been collected, three further sources of auxiliary information are required to provide researchers with the final database. First, WDC format files do not contain any observatory metadata, such as observatory positions in latitude, longitude and altitude. This information is essential if data are to be used for global modelling, for example. We use the WDC metadata web service (British Geological Survey 2025b) to provide this information. The IAGA2002 format files from INTERMAGNET and TGO can contain such metadata in their file headers; however, we rely on the values from the WDC metadata web service in all cases. This covers situations, where the WDC holds data for an observatory that is not in INTERMAGNET or TGO and avoids conflict when discrepancies occur between individual file headers or with the details held in the WDC. Clear discrepancies that have been identified in the process of this work have been corrected, querying the observatory operator for confirmation where necessary.

The second types of auxiliary information required to provide continuous time-series are the documented baseline changes which occur during the operation of an observatory. These are used to remove artificial steps which will be present otherwise, and which can occur for a variety of reasons, such as site moves or instrument changes. The baseline changes are reported as part of the annual means series provided by the observatory operator to the WDC. We access the WDC annual means series and extract all reported baseline changes, keeping the observatory’s identifying three-letter International Association of Geomagnetism and Aeronomy (IAGA) code, date of (baseline) change, and the declination, horizontal field and vertical field change values. We use a subset of the WDC annual mean baseline changes, that has been manually checked for applicability to hourly mean data and updated routinely as part of the AUX_OBS_2 production. It should be noted that not all recorded jumps in the WDC are applied to either AUX_OBS_2 or the monthly means series, if they are seen to be detrimental to producing a coherent timeseries. In the process of these works some unambiguously erroneous baseline change values in the WDC were identified and have been corrected where, e.g., the wrong sign or a transcription error in the file was evident.

The third and final source of information is a “live” reference list of data anomalies maintained at BGS. This database contains the dates of undocumented spikes, periods of contaminated data, and step changes which have been manually identified during various efforts to quality control data sets, notably for the ESA Swarm AUX_OBS_2 product. Where step changes have been identified in recent QD data (as is fairly routine), we write off the post-step data, and reassess it at the next quarterly AUX_OBS_2 processing, to check if it has been rectified and data can be re-included. Similarly, anomalies such as spikes and drifts in the QD data are written off initially and reassessed in future, and retained for definitive data if not rectified by the observatory operator before submission to the WDC.

## Methods

**Fig. 1:**
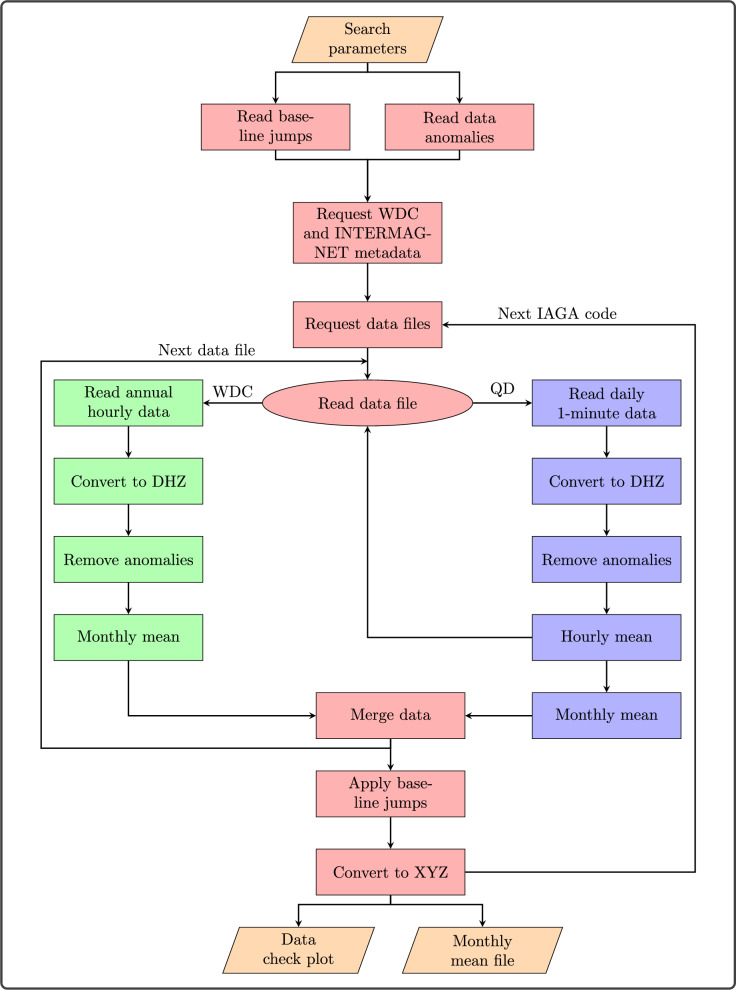
Flow chart of monthly means processing

The processing pipeline consists of several parts including standardising the data into a common form; removing any known anomalous values; merging all data for a given observatory into a single time-series; calculating monthly means; and applying baseline jumps. A more detailed overview of the process is given in Fig. 1.

We choose to convert all observed data to a common intermediate orientation of declination angle (D), horizontal intensity (H), vertical intensity (Z) to match the storage format of the curated baseline jumps used for the AUX_OBS_2 data processing, which has been expanded to cover pre-1900 data for this database. Working in D, H, Z orintation is to avoid the need to extrapolate or interpolate the neighbouring annual means, given at the mid-point of each year, to the year ends, where baseline changes are reported, to compute each field component of the baseline change as a vector addition to the main field vector at that time. Finally, data are converted to (geodetic, according to the WGS84 reference ellipsoid) north (X), east (Y), down (Z) orientation for file output. Due to these conversions, we only report data when there is sufficient information to derive at least one of D, H, Z, and subsequently at least one of X, Y, Z. This includes using reported scalar total field intensity where appropriate.

We found during this work that some observatories update the QD data file they deliver to INTERMAGNET for a given day, and that there can be overlap in time between reported QD data and the definitive data housed in the WDC. We use the most recent QD data file versions available, and only those which do not conflict with any definitive data in the WDC.

The final reported monthly means are rounded to 1 nT precision as provided by the WDC hourly data storage format (British Geological Survey 2025a), and any QD data included is similarly rounded for consistency, though it may have been reported at higher precision.

## File format

We report monthly means in a file format similar to but not exactly the same as Chulliat and Telali (2007) in the previous incarnation of this database. This is a modified form of the IAGA2002 format (IAGA Div. V DAT 2016), with a reduced header, modified data flags, and without a specified record length. As with Chulliat and Telali (2007), we prefer to keep a single file of unspecified length per observatory to accommodate the data available, rather than restrict each file to a single day or year, as a practicality given the century-long duration of some observed time-series and relatively low number of time samples. This allows a more convenient and efficient format for users.

Each file begins with an IAGA2002-style header abbreviated to only the observatory position metadata and any comments, e.g.,
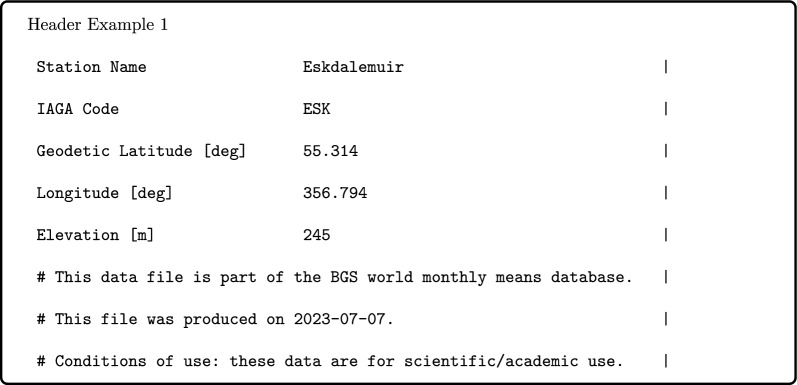


Departing from Chulliat and Telali (2007), we include two additional information sections as comments in the file headers. First, we apply quality control to the reported hourly or minutely data, following the approach described by Macmillan and Olsen (2013) using a database of anomalies created by and held at BGS. Where data have been removed in such a manner, a header comment as in Header Example 2 will be included in the file.
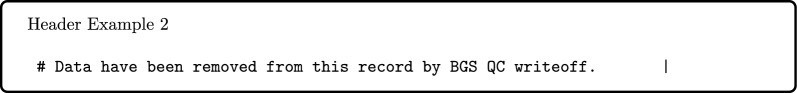


Second, we apply all baseline changes documented by the observers to the data, rather than interspersing the change values in the uncorrected data series. Hence, the header may then include details of baseline changes that have been applied to the data in D, H, Z orientation, e.g.,
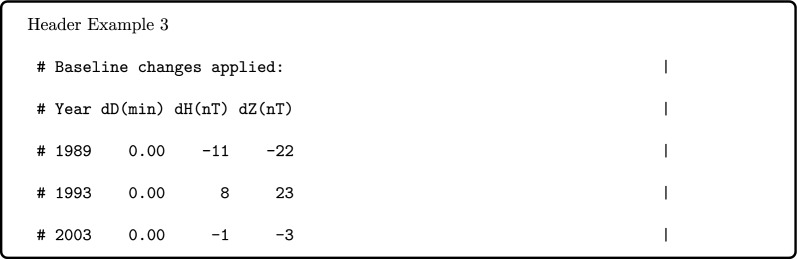
 This part of the header creation is skipped if no baseline changes have been applied. Baseline changes are applied to all data preceding the end of the year specified, as standard, and the units used (minutes of arc and nT) are noted. Applying these changes means the data series are ready for use by researchers, while providing a record of the changes applied helps with questions of data provenance and quality.

Following this is a data header line to indicate the start of the time-series and subsequent data entries, e.g.,
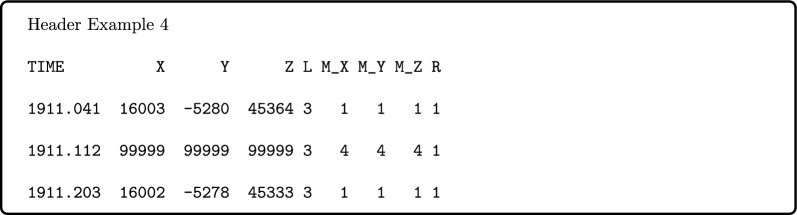
 Monthly data are reported in geodetic X, Y, Z components. There is no specified number of data entry rows, the file will contain rows spanning the first available month of valid data to the last available month of valid data. The monthly data series thus do not necessarily match up with the widest extent of available source hourly and 1-min data files, if the higher cadence data these contain are not valid for any reason. Within the reported valid data span, all possible time samples are given, with fill values of “99999” used to denote missing data, as shown in Header Example 4.

**Fig. 2:**
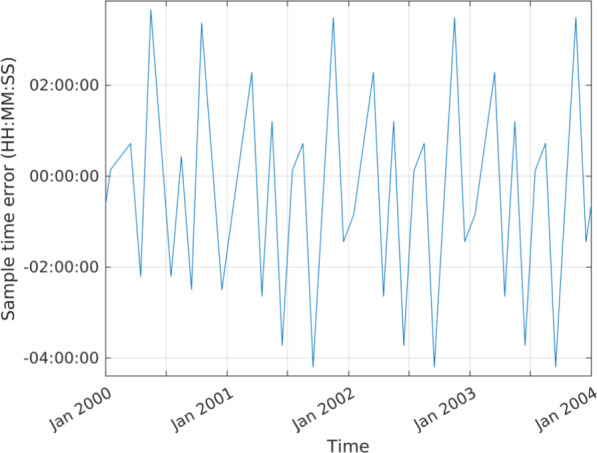
Error in monthly sampling times introduced by abbreviating the precision of decimal year to three decimal places

Monthly data sampling times are described in Table 1. Data are reported at the middle hour of the calendar month, as approximated in decimal years to three decimal places. This means that the sampling is irregular in time due to varying month length and thus also differs for leap years. Where incomplete data sampling is available for a given month, the value or fill is still reported at the standard middle hour, for consistency. The impact of the abbreviation of the reported sample times is illustrated in Fig. 2, where it can be seen that the error is cyclical with the pattern of years and leap years. The difference is approximately within $\pm 4$ h, which is negligible when considering the relatively slow variation of the core field and the monthly averaging applied.

**Table 1:** Example monthly time samples for leap years and non-leap years

365 day year		366 day leap year	
Date-time	Decimal year	Date-time	Decimal year
16-Jan-1982 00:00:00	1982.041	16-Jan-2020 00:00:00	2020.041
14-Feb-1982 12:00:00	1982.122	15-Feb-2020 00:00:00	2020.123
16-Mar-1982 00:00:00	1982.203	16-Mar-2020 00:00:00	2020.205
15-Apr-1982 12:00:00	1982.286	15-Apr-2020 12:00:00	2020.289
16-May-1982 00:00:00	1982.370	16-May-2020 00:00:00	2020.372
15-Jun-1982 12:00:00	1982.453	15-Jun-2020 12:00:00	2020.456
16-Jul-1982 00:00:00	1982.537	16-Jul-2020 00:00:00	2020.539
16-Aug-1982 00:00:00	1982.622	16-Aug-2020 00:00:00	2020.624
15-Sep-1982 12:00:00	1982.705	15-Sep-2020 12:00:00	2020.708
16-Oct-1982 00:00:00	1982.789	16-Oct-2020 00:00:00	2020.791
15-Nov-1982 12:00:00	1982.872	15-Nov-2020 12:00:00	2020.875
16-Dec-1982 00:00:00	1982.956	16-Dec-2020 00:00:00	2020.958

We provide three types of data flags to describe the magnetic element values, similar to those of Chulliat and Telali (2007): “L”, “M_” and “R”, where flag values for each code are described in Tables 2, 3 and 4, respectively.

**Table 2:** Observed element “L” code description

L	Observed elements used to generate monthly mean, might be only some of those reported in the original data file
1	D, H, Z
2	D, H, I
3	X, Y, Z
4	D, I, F
5	D, H, F
6	<3 elements reported, D,H,Z reported directly as available
7	<3 elements reported, D,H calculated from X,Y as available
9	No data reported covering this time, including missing from existing file
0	Data exists but none of the above options (e.g., only F reported), X,Y,Z filled with ‘99,999’ value in output file

**Table 3:** Data availability “M” code description, by component (M_X, M_Y, M_Z)

M_	Monthly means derived from
1	All hours in calendar month
2	Incomplete data ($\ge$50% hours in calendar month)
3	Very incomplete data (<50% hours in calendar month)
4	No valid data in month
9	No data file covering this time

**Table 4:** Source of data “R” code description

R	Source of data (highest value code when data sources combined in a single sample)
1	WDC annual file, hourly mean data,.wdc format
2	INTERMAGNET quasi-definitive IAGA2002 format daily 1-min file
3	TGO quasi-definitive IAGA2002 format daily 1-min file
4	BGS edited WDC annual file, hourly data,.wdc format
9	No data reported covering this time, including missing from existing file

The “L” code describes which magnetic elements originally reported by the observatory we use to produce each monthly mean value, before we standardised them to XYZ. Where multiple values of “L” are present for the hourly or 1-min data values aggregated into a single monthly sample, we report the most common (mode) value corresponding to valid data points.

The “M_” code has been split into three sub-flags compared to Chulliat and Telali (2007): “M_X”, “M_Y”, “M_Z”, to indicate the percentage of hourly or 1-min data available to produce each monthly sample in the relevant magnetic vector element. The flags given for XYZ elements may initially have been derived from equivalent flag values for the originally reported elements if they differed from XYZ. Where this is the case and two elements with different flag values are combined to produce X, Y or Z, the larger of the two associated flag values is retained to indicate the worse selection of data availability is possible.

The “R” code describes the source of the data aggregated for each monthly sample. This can be WDC, INTERMAGNET QD, TGO near-definitive data, a manually edited WDC record, or none if no data are reported for a given time sample with the span of valid reported data. Where multiple data sources are combined to produce a monthly value, the higher code value is given, indicating the lowest quality of data present.

### Data access

The database is updated each month, and the monthly means data, check plots, and acknowledgement text files for each observatory are available through an application programming interface (API) hosted as part of the WDC, with access details given at https://wdc.bgs.ac.uk/monthlymeans/. Only the most recent database version is available through the API, though old versions are archived internally at BGS as a backup. The new WDC API (https://wdcapi.bgs.ac.uk/), provides documented and executable examples of usage, including for the monthly means database. This web service provides access to all data in the WDC, and includes endpoints for the Heliophysics Data Application Programmer’s Interface https://hapi-server.org/ (Weigel et al. 2021). For the monthly means database, users can request data for a specific observatory, or all available data, which is delivered in the designed IAGA2002-style format.

The integration of the full WDC catalogue with an encompassing API, means that the main WDC data portal (https://wdc-dataportal.bgs.ac.uk/) can also be used to view, plot, and download the monthly means data. This allows users to view monthly data availability for a given time period, and download of the data in various additional output formats, such as XML, CSV, or JSON.

## Results

**Fig. 3:**
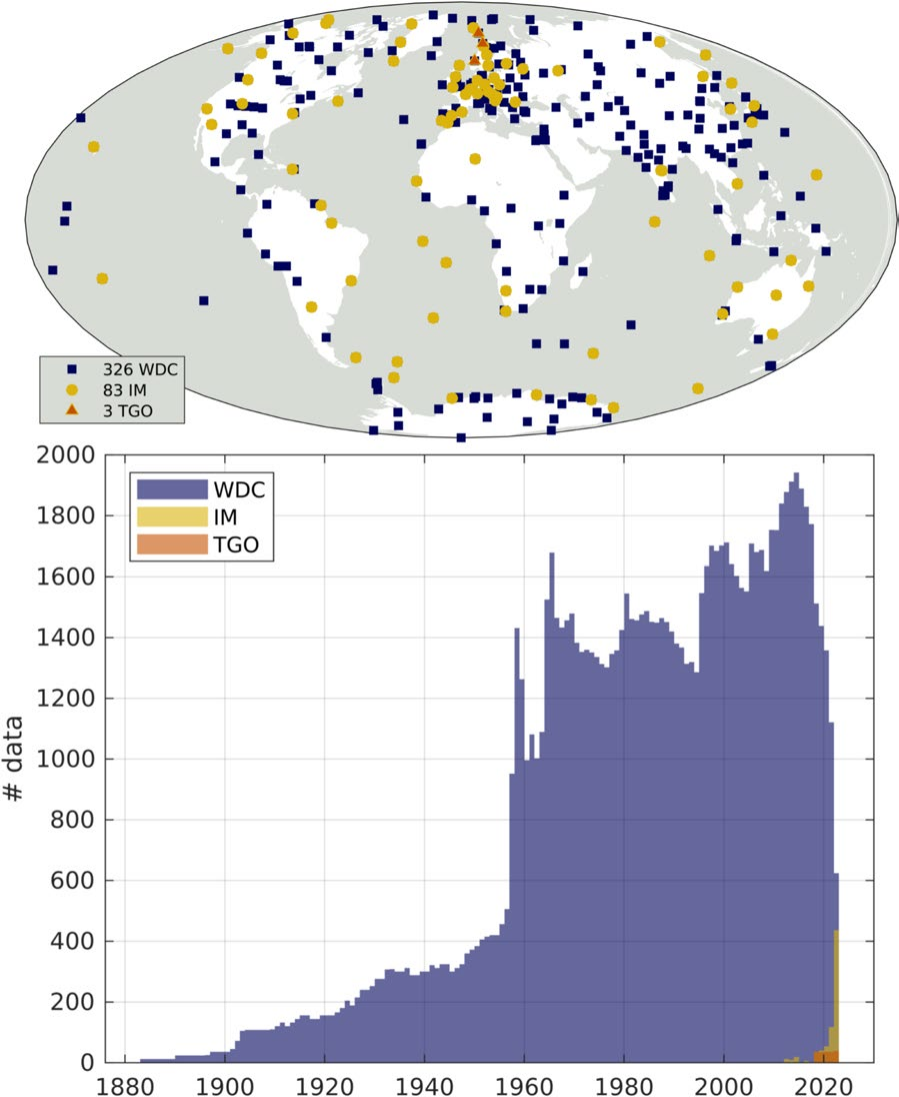
Locations of the 327 observatories providing data for this database (top) and time distribution of the approximately 327,000 monthly samples in the database (bottom), from either hourly means in the WDC, or 1-min quasi-definitive data through INTERMAGNET or TGO. Data are binned by calendar year

We have produced monthly means series for 327 observatories spanning 1883 to the present day, with a total of approximately 327,000 data samples. The distribution of data in time and space is illustrated in (Fig. 3). These series include 83 observatories reporting quasi-definitive data to INTERMAGNET and 3 observatories reporting similar data to TGO, at the time of writing. One observatory is currently reporting quasi-definitive data to INTERMAGNET for which there is not yet definitive data published in the WDC.

**Fig. 4:**
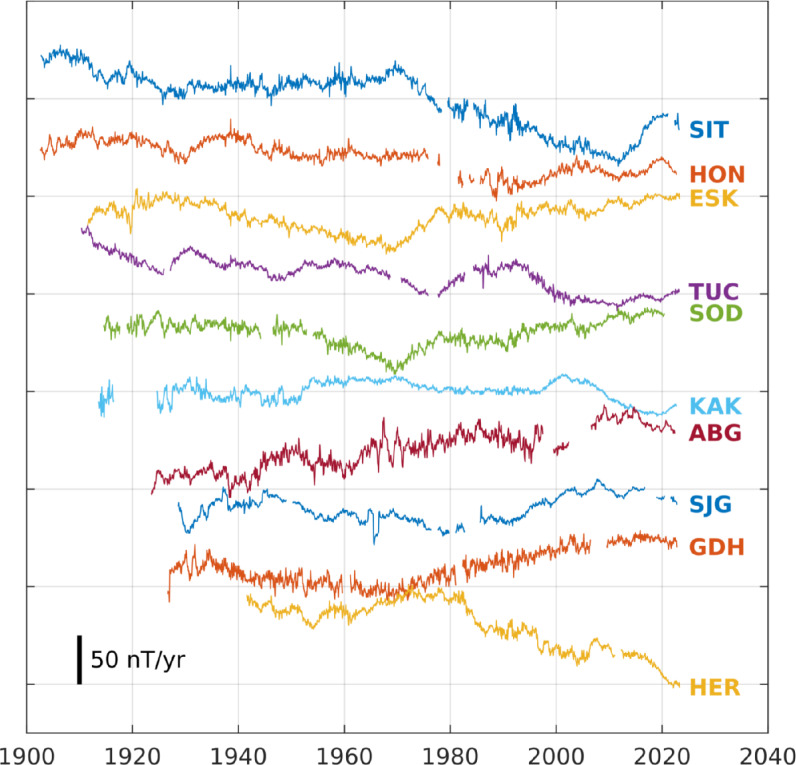
Selected example time-series of Eastward (Y) secular variation of monthly means, in order of most to fewest time samples, from top to bottom. Observatories at: Sitka, Alaska, USA (SIT); Honolulu, Hawai’i, USA (HON); Eskdalemuir, UK (ESK); Tucson, Arizona, USA (TUC); Sodankyla, Finland (SOD); Kakioka, Japan (KAK); Alibag, India (ABG); San Juan, Puerto Rico (SJG); Godhavn, Greenland (GDH); Hermanus, South Africa (HER)

Figure 4 shows examples of SV calculated from the monthly means series produced for the Eastward (Y) component. The SV was calculated as annual differences of the monthly means. There are six observatories with over 100 years worth of non-consecutive data: Sitka, Alaska, USA (SIT); Honolulu, Hawai’i, USA (HON); Eskdalemuir, UK (ESK); Tucson, Arizona, USA (TUC); Sodankyla, Finland (SOD); Kakioka, Japan (KAK). ESK is (at time of writing) the sole observatory with a continuous record of 100 years of monthly data. The remaining four observatories shown in Fig. 4 provide some of the longest, most complete records outside of Europe and the continental US.

## Discussion

### Corrections to data files

The process of deriving initial database files led to correction of various errors in file names, formats and contents of files housed within the WDC. In accordance with the policy of the WDC of archiving and making available the data submitted by observatory operators unaltered, no arbitrary corrections to the original data values were made. The exception to this was where clear (presumably human) typos were identified. For example, a file purporting to contain data from 1962 may contain a limited number of entries which are reported for transposed year “26”, rather than “62”. Similarly, a single day row of data from mid-year may be present with a tabular base value that is not consistent with the otherwise constant base value given for the rest of the entries.

For INTERMAGNET QD files, no alterations were made. We note two comments on the content of these files. First, that metadata on observatory position is not always included and not always consistent between files for a single observatory; hence, we rely on the single reference point of the WDC metadata web service for this information. Second, that variation files are occasionally reported as QD files, or the header values of reported geomagnetic variation elements which differ from those of absolute elements are sometimes given. In such cases it could not be definitively identified what elements were reported, and such files were ignored.

### Data precision

WDC hourly files provide data to 1 nT, but INTERMAGNET files in IAGA2002 format allow (though do not require) greater precision to be reported. We choose to report monthly mean data derived from both types of source file at 1 nT precision, both for consistency and as recognition of the impact on precision of taking a mean over a relatively long period. It would be possible in some cases to return to original files other than the WDC annual files of hourly means to obtain historic values at higher cadence and precision. The benefit of this would be limited in terms of availability of data, and largely meaningless in terms of precision gained in temporal-average values representing predominantly the relatively slow varying field from the core. A 1 nT precision is around 1 part in 50,000 for the average core field which over a period of one century represents only a small part of the overall uncertainty associated with the measurements. If one were to derive the annual SV from the monthly mean values, then 1 nT precision will become a more significant factor; roughly 1 part in 50 of the signal remaining. The variance of the data over a month is typically an order of magnitude greater than 1 nT, with external fields which may vary by up to thousands of nanoTesla within a day during disturbed periods, and so 1 nT precision is likely still over-precise considering the uncertainty on each monthly datum.

### Quality control

Quality control (QC) of geomagnetic observatory data is a particularly difficult task to perform objectively. The ideal QC procedure would be cognisant of factors such as variation in geomagnetic environment by geographic location and quality of data expected based on instrumentation used and era of recording (e.g., digital versus analogue equipment). It should be able to identify spikes, steps and gaps in the data, and identify when these are above the expected or accepted level of variation for a given instrument, location, time, and geomagnetic activity level. It should also be able to apply such checks variably throughout time at an observatory, but not be blinkered by focusing only on small windows of data out of context of the full series which may be many decades in length. Given these considerations, a fully automated QC procedure is a complex endeavour. Indeed it takes a human many years of experience to determine whether an anomalous-looking spike or step is artificial or natural.

Various types of whole-series and sliding-window outlier and step-change statistical detection methods were tested. We considered the monthly time-series, their time derivatives and their de-trended residuals as compared to core field models. No method was found to be consistently applicable to all series without subjective input from a user. There is a greater risk of false negatives in longer, quieter data and of false positives in shorter, noisier data. Instead we opt for a ‘light touch’ approach to QC. The above-mentioned QC methods helped to highlight poorer records which were then manually checked in detail. Spot checks were made to compare to existing versions of files from the BCMT database to confirm consistent results for equivalent periods of definitive data.

**Fig. 5:**
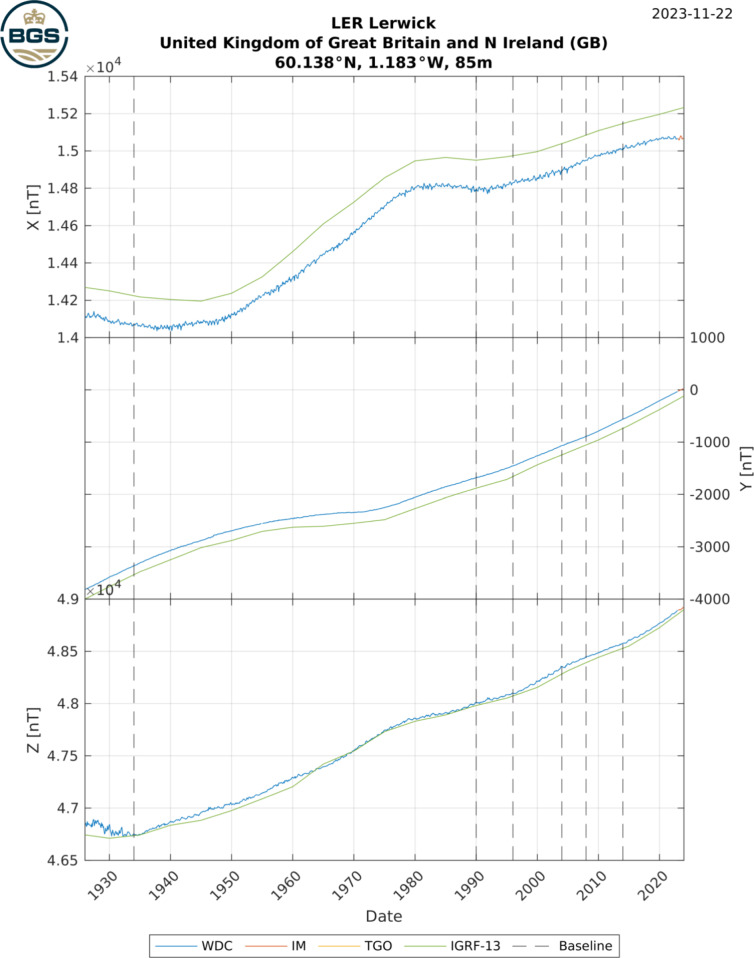
Example check-plot for Lerwick observatory, UK, showing WDC and INTERMAGNET data, IGRF model, and times of applied baseline changes

Check-plots are produced for every series, showing the final complete series compared to the IGRF (for data from 1900–present Alken et al. 2021, ) and GUFM (for data from 1800s Jackson et al. 2000) internal field models, and highlighting the eras of all applied baseline changes as well as the source (WDC, INTERMAGNET, or TGO) of the original data. These checkplots are included in the database and an example is given in Fig. 5. Note that an offset is expected between field model and observatory data series as the models shown represent the core field and omit other field sources, particularly the static crustal field contribution.

As mentioned in the Methods and File format sections, data anomalies are removed during our processing. We have expanded and applied the database of anomalies built over a decade of producing the ESA Swarm Auxiliary Observatory data products “AUX_OBS” ( Macmillan and Olsen (2013), data available at 1-s, 1-min and 1-h cadence, from https://auxobs-api.bgs.ac.uk/). Obviously anomalous data, often artificial spikes or short periods of high frequency and amplitude noise, are removed at a minimum duration of 1 day. Such anomalies are noted by comparing the content of contemporaneous data from other observatory sites, both those geographically close by and those in similar geomagnetic environments, to distinguish, e.g., spikes from external field activity and those from local anthropogenic contamination. This anomaly database is maintained for quasi-definitive data, and for definitive data, with anomalies being discarded or retained as data moves to definitive status, as required. We do not make efforts to split the series from a single observatory about undocumented steps in the data, as is performed for the hourly AUX_OBS_2 data set. Despite these efforts, spikes, steps, gaps, and the effects of contaminating noise remain in some records, and further efforts to extend the anomaly database to exclude these will continue.

Rather than make our own judgement as to the utility of a given record based on length or quality of record, we have endeavoured to include all observatories which can provide any monthly data. We recommend that users consult the check plots for a summary of the data at each observatory before performing their own studies. For studies of the SV, our recommendation would be to use records of at least 3 year length, so as to have two full cycles of annual differences for robustness. Typically further effort than just QC is required to separate the internal field signal from the external field “noise” for internal field modelling purposes, and more intensive schemes (e.g., Cox et al. 2018) can be applied.

### Digitisation of early Greenwich observatory monthly means, 1841 to 1925

With the aim of extending and expanding the catalogue of data available from the 1800s, recent conservation activities at BGS included assessing digital scans of physical yearbooks in our collections from historic UK observatories. Using the scanned Royal Observatory, Greenwich (GRW, 51$^\circ$29’N 0$^\circ$0’E) yearbooks spanning 1841 to 1925 (British Geological Survey 2022a), the monthly mean values of declination, inclination, horizontal force and vertical force were digitised (Maume et al. 2023; British Geological Survey et al. 2025). Monthly declination and inclination records are mostly complete and straightforward to translate from this period, but interpreting the monthly horizontal and vertical force measurements is more complicated. The latter were recorded in various non-SI units, require correction for temperature (as per temperature values also recorded by the observers), and prior to 1912 also require a diminishing scale factor to be recalculated to adjust the monthly values recorded. The technical challenge of translating the pre-1912 force observations is documented in Maume et al. (2023), and could not be conclusively determined, and so the force values will not be discussed further in this report. A separate effort undertook digitisation of daily magnetograms from UK observatories at minute resolution for major historic geomagnetic storms over the past two centuries (Beggan et al. 2023), and records of the 1859 Carrington storm specifically (Beggan et al. 2024).

The yearbooks varied in size from 70 pages to 648 pages and data from multiple readings of multiple instruments were digitised, taking between 1 and 5 h of effort per yearbook. The “raw” data as given in the yearbooks were digitised manually, and subsequent calculations were made to convert the monthly mean declination and inclination from degrees and decimal minutes or degrees, minutes and seconds, to decimal minutes to ensure homogeneous units through time. The annual means reported in the log books were similarly digitised to give a series of validation points to highlight transcription errors, and to compare to the annual means held in the WDC already, which were complied by various earlier works from the same yearbooks (notably, Malin and Bullard 1981).

**Fig. 6:**
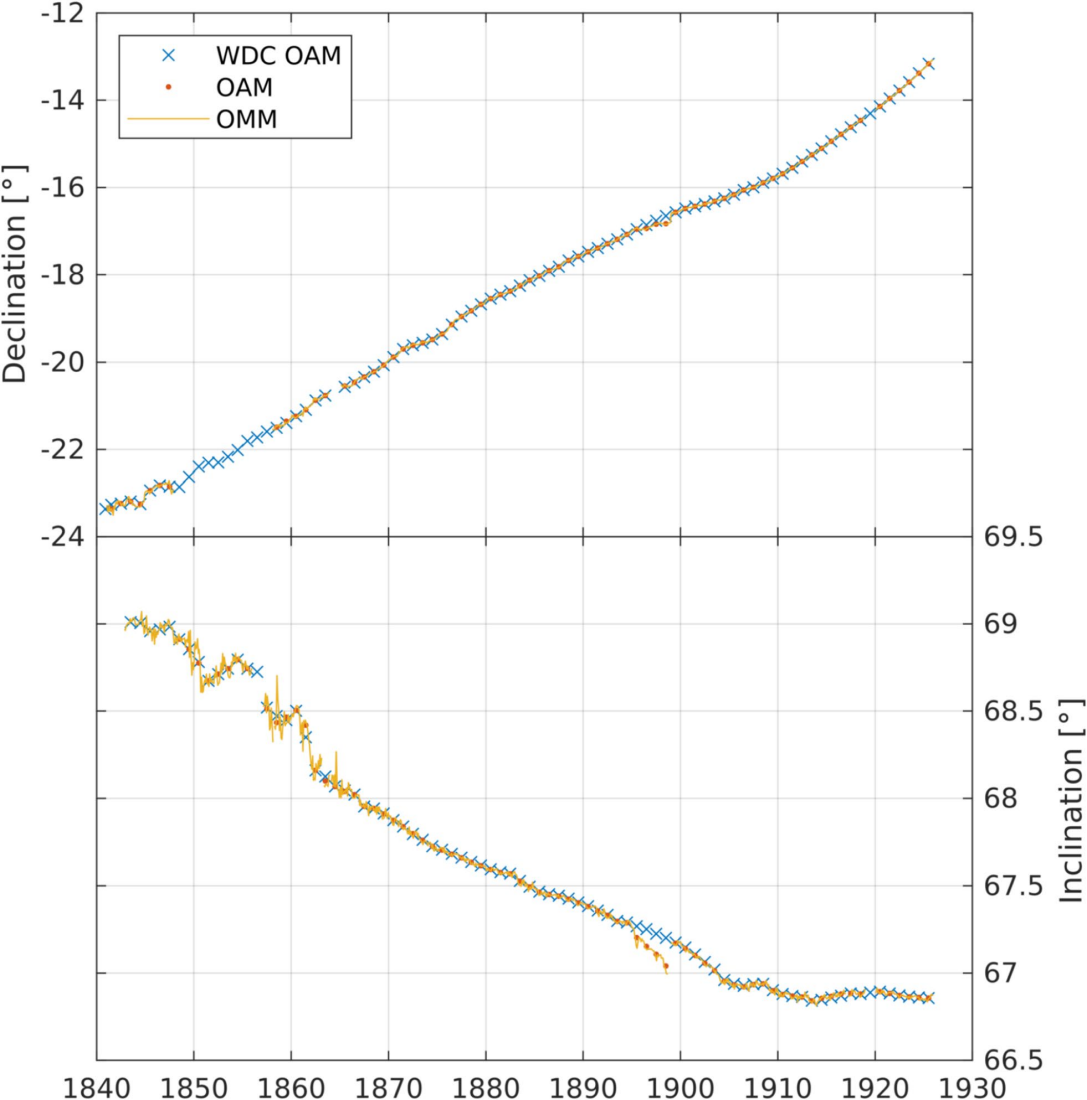
Observatory annual means (OAM) and monthly means (OMM) of digitised declination (top) and inclination (bottom) values from Greenwich yearbooks from 1841 to 1925, and corrected annual means held by the World Data Centre

The monthly mean declination and inclination values are shown in Fig. 6. There are some gaps in the records. There are no monthly declination readings reported: between 1848 and 1857; for 1864; for 1919, as the yearbook digital scan is not available. There are no monthly inclination readings reported: prior to November 1842; for 1856; for March 1858; for March–April 1863; for January 1864; for October–December 1898, January–February 1917; for 1919. The values for 1895 to 1899 of both declination and inclination are affected by construction of the east and west wings at the observatory, introducing more iron and steel. As detailed in the 1899 yearbook, corrections to account for this construction were calculated and applied to the annual means from 1899 onward by the observatory, but these corrections are not reported at the monthly cadence. The corrected annual means, reported to the WDC, can be seen in Fig. 6, and compared to the original uncorrected annual means digitised from the yearbooks for this work, which validate the digitised monthly means. Further work is needed to ascertain if monthly corrected data can be calculated from the records.

In 1861, the inclination instrumentation changed from Robinson’s dip apparatus to Airy’s dip apparatus, and the resulting improvement in data quality can be attributed to this. The 1910 yearbook documents a correction to be applied between 1868 and 1910, and continued thereafter, for inclination readings from Airy’s apparatus. This correction accounts for an apparent dip observed due to offset of the dip needle centre of mass and the dip needle pivot. This correction is on the order of arc-seconds and has not been applied to the reported digitised data here; the resulting offset between the uncorrected digitised monthly and annual means and corrected WDC annual means is small enough in Fig. 6 to not be noticeable.

From 1912 to 1925 the monthly mean horizontal and vertical force values were also converted from CGS units into nanoTesla (nT). Before 1912 the monthly horizontal and vertical data were not recorded continuously and the units used, and corrections necessary to convert them to nT, are not clear. Because of the absence of absolute intensity data the Greenwich monthly means could not become part of the monthly means database at this stage; more work is required to achieve this. The Greenwich magnetic observatory was closed in 1926, when the electrification of London’s railways necessitated a move to the newly established Abinger, Surrey (ABN). Abinger was in turn affected by expanding electrified rail, and the observatory subsequently moved to the current BGS observatory site at Hartland, Devon (HAD), in 1957.

The digitised monthly declination, inclination, horizontal force and vertical force values are available via the National Geoscience Data Centre (British Geological Survey et al. 2025) and a full description of the data and its preparation is presented by Maume et al. (2023).

## Summary

We have described the creation of a new database of geomagnetic observatory monthly means, to replace the defunct BCMT World Monthly Means database. Our database combines the complete WDC catalogue of definitive hourly data, as well as recent near-definitive data, and will be updated monthly to keep it as current as possible. 327 observatory records are compiled, of which 86 include near-definitive data. The earliest data are from 1883, with records extending up to the present time. The data are presented in a consistent format, with anomalous data removed, baseline changes applied, and a single merged record for each observatory, for users’ convenience.

Geomagnetic observatory data are a vital source of information on the historic and modern geomagnetic field, particularly the secular variation of the core field. It is hoped that this database will allow researchers to analyses data more easily, and support a variety of studies.

We have additionally reported newly digitised monthly mean values for 1841–1925 from the Royal Observatory, Greenwich, some of the earliest magnetic data at monthly time resolution which may aid historical studies.

## Data Availability

The observatory monthly means database can be found via the homepage https://wdc.bgs.ac.uk/monthlymeans/, and directly by API at https://wdcapi.bgs.ac.uk/. The data from the Royal Observatory, Greenwich is available through the National Geoscience Data Centre as British Geological Survey et al. (2025) and the accompanying report is Maume et al. (2023). Reported observatory data files were sourced from the World Data Centre for Geomagnetism, Edinburgh (https://wdc.bgs.ac.uk), INTERMAGNET (https://intermagnet.org), and Tromsø Geophysical Observatory (https://www.tgo.uit.no).

## References

[CR1] Alken P, Thébault E, Beggan CD, Amit H, Aubert J, Baerenzung J, Bondar T, Brown W, Califf S, Chambodut A et al (2021) International geomagnetic reference field: the thirteenth generation. Earth, Planets Space 73(1):1–25. 10.1186/s40623-020-01288-x

[CR2] Beggan CD, Eaton E, Maume E, Clarke E, Williamson J, Humphries T (2023) Digitizing UK analogue magnetogram records from large geomagnetic storms of the past two centuries. Geosci Data J 10(1):73–86. 10.1002/gdj3.151 37035263 10.1002/gdj3.151 PMC10078682

[CR3] Beggan CD, Clarke E, Lawrence E, Eaton E, Williamson J, Matsumoto K, Hayakawa H (2024) Digitized Continuous Magnetic Recordings for the August/September 1859 Storms From London, UK. Space Weather, 22(3):e2023SW003807, 10.1029/2023SW003807

[CR4] British Geological Survey. Greenwich magnetic observatory yearbooks. https://geomag.bgs.ac.uk/data_service/data/yearbooks/grw.html, 2022a. Accessed: 2022-07-07

[CR5] British Geological Survey. INTERMAGNET data access. https://imag-data.bgs.ac.uk/, 2022b. Accessed 12 Jan 2022

[CR6] British Geological Survey. World Data Centre for Geomagnetism, Edinburgh. https://wdc.bgs.ac.uk/data.html, 2022c. Accessed 7 July 2022

[CR7] British Geological Survey. World Data Centre file format description. https://wdc-dataportal.bgs.ac.uk/(FAQ, The WDC Exchange Format), 2025a. Accessed 10 July 2025

[CR8] British Geological Survey. World Data Centre for Geomagnetism, Edinburgh, web service documentation. https://wdcapi.bgs.ac.uk/docs#/, 2025b. Accessed 10 July 2025July 2025

[CR9] British Geological Survey, E. Maume, E. Eaton, S. Macmillan, and W. Brown. Monthly magnetic data from the Greenwich Magnetic Observatory Yearbooks (1841 – 1925). NERC EDS National Geoscience Data Centre (Dataset), 2025. 10.5285/bc36472c-de05-4981-8c8b-43677ad7f956

[CR10] Brown W, Mound J, Livermore P (2013) Jerks abound: An analysis of geomagnetic observatory data from 1957 to 2008. Phys Earth Planet Inter 223:62–76. 10.1016/j.pepi.2013.06.001. (**SI:13th SEDI Conference**)

[CR11] Chulliat A, Telali K (2007) World Monthly Means DataBase Project. Publ. Inst. Geophys. Pol. Acad. Sci., C-99 (398), URL http://www.bcmt.fr/wmmd.html

[CR12] Clarke E, Baillie O, Reay SJ, Turbitt CW (2013) A method for the near real-time production of quasi-definitive magnetic observatory data. Earth Planets Space 65(11):1363–1374. 10.5047/eps.2013.10.001

[CR13] Cox G, Brown W, Billingham L, Holme R (2018) MagPySV: A Python package for processing and denoising geomagnetic observatory data. Geochem Geophys Geosyst 19(9):3347–3363. 10.1029/2018GC007714

[CR14] Finlay CC, Kloss C, Olsen N, Hammer MD, Tøffner-Clausen L, Grayver A, Kuvshinov A (2020) The CHAOS-7 geomagnetic field model and observed changes in the South Atlantic Anomaly. Earth Planets Space 72(1):1–31. 10.1186/s40623-020-01252-9 10.1186/s40623-020-01252-9 PMC7578192 33122959

[CR15] Fouassier D, Chulliat A (2009) Extending backwards to 1883 the French magnetic hourly data series. In J. Love, editor, Proceedings of the XIIIth IAGA Workshop on geomagnetic observatory instruments, data acquisition and processing, number 1226 in Open-File Report, pages 86–94. U.S. Geological Survey, URL https://pubs.usgs.gov/of/2009/1226/

[CR16] Geophysical Center of the Russian Academy of Sciences. World Data Center for Solar-Terrestrial Physics, Moscow. http://www.wdcb.ru/stp/index.en.html, 2021. Accessed 7 July 2022

[CR17] Graduate School of Science, Kyoto University. World Data Centre for Geomagnetism, Kyoto. https://wdc.kugi.kyoto-u.ac.jp/, 2022. Accessed 7 July 2022

[CR18] IAGA Div. V DAT. IAGA2002 file format description. https://www.ngdc.noaa.gov/IAGA/vdat/IAGA2002/iaga2002format.html, 2016. Accessed 7 July 2022

[CR19] INTERMAGNET. INTERMAGNET homepage. https://intermagnet.github.io/, 2022. Accessed 7 July 2022

[CR20] Jackson A, Jonkers A, Walker M (2000) Four centuries of geomagnetic secular variation from historical records. Philos Trans R Soc Lond Ser A-Math Phys Eng Sci 358(1768):957–990. 10.1098/rsta.2000.0569

[CR21] Macmillan S, Olsen N (2013) Observatory data and the swarm mission. Earth Planets Space 65(11):1355–1362. 10.5047/eps.2013.07.011

[CR22] Malin S, Bullard E (1981) The direction of the earth’s magnetic field at london, 1570-1975. Philosophical Transactions of the Royal Society of London. Series A. Math Phys Sci 299(1450):357–423. 10.1098/rsta.1981.0026

[CR23] Mandea M, Holme R, Pais A, Pinheiro K, Jackson A, Verbanac G (2010) Geomagnetic jerks: Rapid core field variations and core dynamics. Space Sci Rev 155(1–4):147–175

[CR24] Maume E, Eaton E, Macmillan S, Brown W (2023) Digitising historic datasets: Greenwich Magnetic Yearbooks 1841-1925. Technical Report OR/23/061, British Geological Survey, URL https://nora.nerc.ac.uk/id/eprint/536965/

[CR25] Peltier A, Chulliat A (2010) On the feasibility of promptly producing quasi-definitive magnetic observatory data. Earth Planets Space 62(2):e5–e8. 10.5047/eps.2010.02.002

[CR26] Tromsø Geophysical Observatory. TGO data access. https://flux.phys.uit.no/geomag.html, 2022. Accessed 7 July 2022

[CR27] Weigel RS, Vandegriff J, Faden J, King T, Roberts DA, Harris B, Candey R, Lal N, Boardsen S, Lindholm C, Lindholm D, Baltzer T, Brown LE, Grimes EW, Cecconi B, Génot V, Renard B, Masson A, Martinez B (2021) HAPI: An API Standard for Accessing Heliophysics Time Series Data. J Geophys Res: Space Phys. 10.1029/2021JA029534

